# Amplitude of fNIRS Resting-State Global Signal Is Related to EEG Vigilance Measures: A Simultaneous fNIRS and EEG Study

**DOI:** 10.3389/fnins.2020.560878

**Published:** 2020-12-03

**Authors:** Yuxuan Chen, Julia Tang, Yafen Chen, Jesse Farrand, Melissa A. Craft, Barbara W. Carlson, Han Yuan

**Affiliations:** ^1^School of Electrical and Computer Engineering, University of Oklahoma, Norman, OK, United States; ^2^Stephenson School of Biomedical Engineering, University of Oklahoma, Norman, OK, United States; ^3^Fran and Earl Ziegler College of Nursing, University of Oklahoma Health Sciences Center, Oklahoma City, OK, United States; ^4^Institute for Biomedical Engineering, Science, and Technology, University of Oklahoma, Norman, OK, United States

**Keywords:** electroencephalography, functional near-infrared spectroscopy, resting state, vigilance, global signal

## Abstract

Recently, functional near-infrared spectroscopy (fNIRS) has been utilized to image the hemodynamic activities and connectivity in the human brain. With the advantage of economic efficiency, portability, and fewer physical constraints, fNIRS enables studying of the human brain at versatile environment and various body positions, including at bed side and during exercise, which complements the use of functional magnetic resonance imaging (fMRI). However, like fMRI, fNIRS imaging can be influenced by the presence of a strong global component. Yet, the nature of the global signal in fNIRS has not been established. In this study, we investigated the relationship between fNIRS global signal and electroencephalogram (EEG) vigilance using simultaneous recordings in resting healthy subjects in high-density and whole-head montage. In Experiment 1, data were acquired at supine, sitting, and standing positions. Results found that the factor of body positions significantly affected the amplitude of the resting-state fNIRS global signal, prominently in the frequency range of 0.05–0.1 Hz but not in the very low frequency range of less than 0.05 Hz. As a control, the task-induced fNIRS or EEG responses to auditory stimuli did not differ across body positions. However, EEG vigilance plays a modulatory role in the fNIRS signals in the frequency range of less than 0.05 Hz: resting-state sessions of low EEG vigilance measures are associated with high amplitudes of fNIRS global signals. Moreover, in Experiment 2, we further examined the epoch-to-epoch fluctuations in concurrent fNIRS and EEG data acquired from a separate group of subjects and found a negative temporal correlation between EEG vigilance measures and fNIRS global signal amplitudes. Our study for the first time revealed that vigilance as a neurophysiological factor modulates the resting-state dynamics of fNIRS, which have important implications for understanding and processing the noises in fNIRS signals.

## Introduction

Functional near-infrared spectroscopy (fNIRS) is a non-invasive functional neuroimaging technique that can monitor concentration changes in oxygenated and deoxygenated hemoglobin (HbO and HbR) in the cerebral cortex. fNIRS measurement is based on the absorption of light in near-infrared spectrum from 700 to 1000 nm by biological tissues. Different chromophores, such as hemoglobin, myoglobin, and cytochrome aa3, have different absorptivity (Sood et al., 2015). With the advantage of low-cost, portability, and ease to co-register with other neural recording modalities, such as an EEG and fNIRS has become an attractive means for imaging and monitoring hemodynamic signals in the human brain, which complements the use of fMRI in versatile environment. fNIRS has been widely applied in functional neuroimaging (Torricelli et al., 2014; Chen et al., 2020), cerebral monitoring in neonates (Sood et al., 2015; Hu et al., 2020) and brain-computer interface (Naseer and Hong, 2015; Ahn and Jun, 2017; Shin et al., 2017). Unlike fMRI constraining subjects to lying down on a scanner bed, fNIRS poses fewer physical constraints on the participants, thereby permitting them to be studied at flexible body positions during recordings.

Particularly, imaging of resting-state functional connectivity (RSFC) in the human brain has been a recent focus for neuroimaging studies, including fNIRS (Mohammadi-Nejad et al., 2018; Pinti et al., 2018). The activity of the resting brain exhibits spontaneous and large-amplitude fluctuations, which have been observed in a number of imaging modalities such as fMRI (Biswal et al., 1995), positron emission tomography (Raichle et al., 2001; Watabe and Hatazawa, 2019), and direct measures of neuronal activity with electro- or magneto-encephalography (EEG or MEG) (Goldman et al., 2002; Mantini et al., 2007; Brookes et al., 2011; Yuan et al., 2012, 2016). The measures of resting-state cerebral hemodynamics, mostly using fMRI based on the blood-oxygenation-level dependent (BOLD) contrast, show fluctuations predominantly at a low frequency band of <0.1 Hz (Cordes et al., 2001). The temporal synchrony across brain regions have been revealed (Beckmann et al., 2005; Damoiseaux et al., 2006), and demonstrated to be important biomarkers for the brain at diseased conditions (Zhang and Raichle, 2010). Prior studies of RSFC in both healthy and diseased conditions can be influenced by the presence of a strong global component, which is usually observed throughout sampled voxels or sensors, thereby dominating the RSFC (Greicius et al., 2003; Fox et al., 2005, 2009). However, the approach of removing global signal has recently been shown to induce systematic biases and the anti-correlation enhanced by global signal regression (GSR) becomes the main concern (Fox et al., 2009; Murphy et al., 2009). Furthermore, evidences show that a neural component (Scholvinck et al., 2010; Wong et al., 2013, 2016) and even diagnostic information (Hahamy et al., 2014; Murphy and Fox, 2017; Yang et al., 2017) exist in the global signal, which challenges the assumption of removing it in the first place.

Like fMRI signals, fNIRS also offers the potential to examine the human brain at resting state by measuring concentration changes of HbO and HbR in the vasculature of the cortical tissues below sensing channels (Obrig and Villringer, 2003; Scholkmann et al., 2014). fNIRS has been effectively employed to characterize the resting-state brain in adults (Obrig et al., 2000; White et al., 2009; Lu et al., 2010; Mesquita et al., 2010; Zhang et al., 2010; Sasai et al., 2011; de Souza Rodrigues et al., 2019), infants or children (Homae et al., 2010; White et al., 2012; Molavi et al., 2013; Watanabe et al., 2017; Bulgarelli et al., 2019, 2020; Wang et al., 2020), and to assess differences between experimental groups (Keehn et al., 2013; Ieong et al., 2019; Arun et al., 2020). The most common RSFC analysis of fNIRS data involves evaluating the temporal relationship between time series of the preprocessed data from recording units, for example, through the Pearson’s correlation. A global component has been observed in fNIRS measurements and commonly removed for the purpose of attenuating systematic noises at the resting state (White et al., 2009; Gregg et al., 2010; Mesquita et al., 2010; Eggebrecht et al., 2014; Tachtsidis and Scholkmann, 2016; Duan et al., 2018; Sherafati et al., 2020; Wyser et al., 2020). Whereas removing superficial contributions from short-distanced channels to fNIRS is increasingly employed to attenuate the systematic noises (Saager and Berger, 2005; Gagnon et al., 2011), data from both long-distanced and short-distanced channels commonly suggest a global component exist in fNIRS measurements and distribute across wide regions (Zhang et al., 2005, 2007, 2009; Kohno et al., 2007; Gregg et al., 2010; Tong and Frederick, 2010; Novi et al., 2016; Sato et al., 2016). However, the physiological nature of the fNIRS global signal has not been fully established, since the neurophysiological components in the resting-state global fNIRS signal have not been systematically investigated. Therefore, whether or not to remove the global signal in fNIRS-based RSFC analysis remains not clear.

In this study, we aimed to investigate the physiological underpinning of resting-state fNIRS global signal by concurrently acquiring fNIRS and EEG in whole-head, high-density montage. Previous studies using simultaneous EEG and fMRI recordings have revealed a negative association between the amplitude of resting state fMRI global signal and EEG vigilance level (Wong et al., 2013; Chang et al., 2016; Falahpour et al., 2018). These studies have shown that subjects at lower vigilance states are characteristic of higher global signal amplitudes, indicating that neurophysiological covariates exist in the global signal. Moreover, the temporal fluctuations of vigilance levels has been linked to the spontaneous activities in regions constituting the default mode network (DMN) (Olbrich et al., 2009) and also linked to the fluctuations of fMRI global signal (Chang et al., 2016; Falahpour et al., 2018), suggesting that regressing out the resting-state global signal could potentially impact the dynamic connectivity in resting state networks. Based on the prior studies using BOLD fMRI, in the current study we hypothesize that the fNIRS global signal has a neurological component and is related to the EEG vigilance. Two experiments were included: the 1st is a 10-min resting study from which we calculated the stationary metrics; the 2nd is a 45-min resting study from which we examined the epoch-to-epoch dynamics during the wakeful epochs. Furthermore, considering that fNIRS is a promising technology for imaging the human brain at versatile body positions, Experiment 1 also examined whether and how different body positions affect the fNIRS global signal at resting state conditions and, as a control, the impact of body positions on evoked activities to auditory stimuli was studied. To our knowledge, our study is the first of its kind to examine the relationship between fNIRS global signal and EEG vigilance with an advanced simultaneous EEG and fNIRS system in high-density and whole-head montage.

## Materials and Methods

### Protocol

All study procedures were completed according to the Declaration of Helsinki guidelines and approved by the Institutional Review Board at the University of Oklahoma Health Sciences Center.

#### Experiment 1: 10-min Resting at Different Body Positions

Twenty-four healthy subjects were recruited after giving informed consent. All subjects were right-handed. Two subjects were excluded due to excessive movements during the recording. Another three subjects were excluded due to high electrode impedance in recording sessions. Thus, nineteen subjects’ data were included in the analysis (11 males and 8 females, aged 19 to 55 years old, average age ± STD = 30.8 ± 12.2 years). Each subject participated in two separate sessions, eyes-open (EO) and eyes-closed (EC), the order of which was randomized. For each subject, two sessions occurred on different days that were within a 4-week period (mean interval ± STD = 18.2 ± 29.5 days). Each session contained three recording blocks at different body positions: standing, sitting, and supine. The order of these blocks was randomized among all subjects but was kept the same for each subject at their consecutive visits. Each block contained a 10-min resting-state part and an auditory task part that lasted 6 min and 30 s. In the resting-state part, subjects were instructed to keep as still as possible and not fall asleep. Specifically, in EO resting condition, subjects were instructed to focus on a black cross on a white background. In the auditory task part, subjects were instructed to keep still and listen to the auditory stimuli from a pair of earbuds. In terms of the presentation, periods of 30-s stimuli on and 30-s stimuli off were interleaved. Within a period of stimuli on, subjects listened to a sequence of 15 brief one-kilohertz tones. One tone lased for 100 ms duration and sampled at 44.1 kHz. Tones within a stimuli-on period were separated by an inter-stimulus interval of 2 s. Six datasets (two eye conditions by three body positions) were obtained for each subject, yielding a total of 114 datasets in the current study, which included concurrent EEG and fNIRS data of both resting state and task conditions.

#### Experiment 2: 45-min Resting at Supine Position

Because Experiment 1 used a stationary design of 10-min resting study to investigate the fNIRS resting state signals, we further included Experiment 2 in the design of a 45-min resting study to examine the temporal dynamics in fNIRS global signal. Specifically, subjects were instructed to rest still and allowed to fall asleep during a 45-min recording, while subjects laid supine in an adjustable recliner. A total of 20 healthy subjects (sex: 12 females and 8 males; aged 28 to 63 years old; average age ± STD: 42.8 ± 11.7 years) were studied in Experiment 2 and no subjects overlapped in Experiments 1 and 2. The recording began and ended with bio-calibration, which were used to identify artifacts in the EEG recordings. The bio-calibration procedure was performed in a standard order of instructing subjects to (1) open and close their eyes, (2) blink, (3) perform lateral eye movements, (4) take deep breaths, (5) clench their teeth, and to (6) speak.

### fNIRS Data Acquisition

An identical configuration of acquisition was used in Experiments 1 and 2. The fNIRS measurements were acquired with a NIRScout system (NIRx Medical Technologies, LLC, New York, United States). Thirty-two source probes and 32 detector probes were plugged into holders and arranged into a cap based on the international 10–5 system (Jasper, 1958). A total of 105 channels (i.e., 105 pairs of sources and detectors) were defined, covering the areas from the forehead to the occipital lobe. The inter-optode distance varied between 25, 27, and 30 mm, corresponding to three different sizes of caps (54, 58, and 60 cm). The intersection between the left and right tragus and the Nasion and Inion was the center of the cap, which was denoted by the Cz position. A dark black over-cap covered the cap to block external light luminance. The absorption of near-infrared light of two different wavelengths (760 and 850 nm) was measured with a sampling rate of 1.95 Hz.

### EEG Data Acquisition

A 64-channel, fNIRS-compatible EEG system (BrainProducts, München, Germany) was utilized to record the EEG data. In order to couple the EEG signal with the fNIRS hemodynamic signal, the montage of the EEG electrodes was designed to match the fNIRS montage. Every EEG channel was crossed by an adjacent pair of light source and detector. Sixty-four EEG electrodes were also mounted onto corresponding holders. The electrode at FCz position was selected as the reference point. Two 32-channel amplifiers, which were powered by a rechargeable battery, were included in our EEG system. Electrically conductive gel was added to decrease the impedance between scalp and electrodes. The impedances of EEG electrodes were kept under 20 kΩ throughout the recordings. All the EEG datasets were digitized with a wide band of 0.1–250 Hz at a 500 Hz sampling rate.

### fNIRS and EEG Data Pre-processing

**FIGURE 1:**
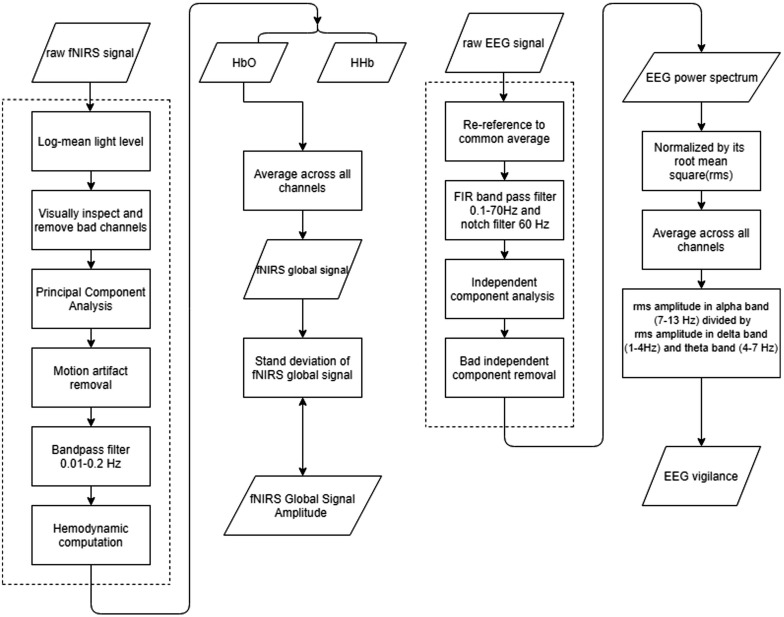
Flowchart of the data processing flowchart. (Left) fNIRS signal processing procedures, and (Right) EEG signal processing procedures. Dashed line circles the pre-processing procedures.

Figure 1 shows the analysis flowchart of EEG and fNIRS data. EEGLAB (Delorme and Makeig, 2004) was used for pre-processing of EEG data. After loading the raw datasets, the data was re-referenced to the common average reference. A basic FIR bandpass filter from 0.1 Hz to 70 Hz was used to filter the data in addition to a notch filter of 60 Hz. Additional ocular and muscular artifacts were removed by the independent component analysis implemented in EEGLAB. The ocular components, muscle movement components, and other artifacts were manually inspected and removed (Chaumon et al., 2015). Preprocessed EEG data were down-sampled to 250 Hz.

fNIRS data was pre-processed in HOMER2 (Huppert et al., 2009). Channels consisted of a source electrode and adjacent detector electrodes. Montages were created according to the setup of sources and detectors. Preprocessing of fNIRS data included converting raw light intensity to optical density, principle component analysis (PCA) removal, and motion artifact detection and correction. The PCA algorithm we performed here is to filter out the first principal component (Novi et al., 2016). Discontinuities and spikes existing in recordings were replaced by an average of its adjacent data segment. All channels were bandpass filtered from 0.01 to 0.2 Hz. The resulted time series were subject to hemodynamic computation via the modified Beer-Lambert law (Kocsis et al., 2006), yielding relative changes in concentrations of Oxy-Hemoglobin (HbO) and Deoxy-Hemoglobin (HbR) (Gratzer and Allison, 1960; Putze et al., 2014).

### Calculation of Resting-State fNIRS Global Signal

After pre-processing, the fNIRS data became a measure of the relative concentration changes of HbO and HbR in units of μM. Then the preprocessed fNIRS were separated into two frequency bands: the lower range of <0.05 Hz and the upper range of >0.05 Hz that contains the Mayer wave (Julien, 2006), guided by inspection of power spectrum and analysis of variance (ANOVA) tests. To calculate the global signal, the time series of relative changes in HbO or HbR were averaged across all channels covering the whole brain. Then, the amplitude of the global signal was defined by the standard deviation of the global signal time series.

### Quantification of EEG Vigilance Level

For EEG data, after removing artifacts, a spectrum was calculated by using Welch’s power spectral density estimate with segments of 10 s and 50% overlap for each EEG channel. Then the spectrum was divided by its overall root mean square (RMS) amplitude of all frequency bins, resulting in the relative amplitude spectrum. The relative amplitude spectrums were then averaged across all channels. Three frequency bands (delta: 1 – 4 Hz, theta: 4 – 7 Hz, alpha: 7 – 13 Hz) were delineated, and the RMS amplitudes were calculated separately for each band. A measure of EEG vigilance was defined as the RMS amplitude in the alpha band divided by the sum of RMS amplitudes in the delta and theta bands (Horovitz et al., 2008; Wong et al., 2013), which is equivalent to the alpha slow-wave index (ASI) (Jobert et al., 1994).

### Auditory fNIRS and EEG Data

Whereas a key investigation of the Experiment 1 was to examine the impact of body position on resting-state neural recordings, we also included investigation of the body position on task-induced responses in EEG and fNIRS, in order to control systematic and environmental nuisances. A mixed block and event-related design were used for the concurrent EEG and fNIRS recordings. One session contained six task blocks and each block contained 15 auditory stimuli. The auditory stimuli were controlled by E-Prime software (Psychology Software Tools, Pennsylvania, United States). Stimuli was sent to earbuds by the stimulation computer. The trigger pulse corresponding to the sound then marked EEG and fNIRS synchronously via a parallel control box. There was a total of six task conditions (standing/sitting/supine body positions X EO/EC conditions).

For fNIRS analysis, block average was obtained after preprocessing in the same way described in resting data. The first marker of a task block was kept as the start of each task block. Based on all available auditory markers (as time 0 s), the time series were demeaned with reference to the time window from −5 to 0 s and averaged, resulting in the auditory response waveform. Segments containing detected motion artifacts were excluded from the average. To visualize the time courses of hemodynamic responses, the fNIRS auditory response was plotted from −10 to 50 s.

For EEG analysis, auditory evoked potentials (AEP) were obtained. Specifically, the recordings were band-pass filtered between 0.1 and 30 Hz, down sampled to 250 Hz, referenced to common average reference, and segmented into epochs from −100 to 500 ms. For every single segment, the *t* = 0 s denotes the onset of auditory stimuli. The mean of the baseline (averaged from −100 to 0 ms) was subtracted from the time series. Ocular and muscular artifacts were removed by the independent component analysis implemented in EEGLAB. Visual inspection further excluded the trials containing motion artifacts. Remaining trials of EEG epochs were averaged for each auditory task condition, resulting the AEP waveforms.

### Sleep Stage Scoring

In Experiment 2, the 45-min recording was reviewed and manually scored into sleep stages by a certified expert (BWC), using standard scoring criteria by the American Academy of Sleep Medicine (Berry et al., 2017). Briefly, EEG data were first segmented into epochs of 30-s length. Based on the frequency and amplitude of the signal, each segment was assigned as awake, non-rapid-eye-movement sleep, Slow Wave Sleep, or rapid-eye-movement sleep. Only epochs of awake stages before first sleep onsite were included in the analysis.

### Statistical Analysis

In Experiment 1, in order to explore the effect of body position on neural recordings (i.e., standing, sitting and supine), ANOVA was applied on the EEG or fNIRS quantities, separately for the EO and EC conditions. We performed the statistical test on each frequency bin along a continuous spectrum (Figures 3, 4). Then, based on the delineation of frequencies, we segregated the quantities of the fNIRS global signal in two bands: *f* < 0.05 and *f* > 0.05 Hz.

Next, two-way repeated measures ANOVA (standing/sitting/supine body positions X EO/EC) was applied to assess if any main effect of body position or eye condition, or interaction between the body position and the eye condition, separately in the frequency range of <0.05 and >0.05 Hz and separately for HbO and HbR. Likewise, two-way repeated measures ANOVA (supine/sitting/standing body positions X EO/EC) was tested on the EEG vigilance scores. Furthermore, *post hoc* analysis assessed the difference between conditions using a paired, two-sided *t-*test. Bonferroni correction was used to correct the multiple comparison problem.

After delineating the position and eye effects, we assessed whether fNIRS global signal amplitudes were associated with the EEG vigilances. Particularly, motivated by a negative relationship between fMRI global signal and EEG vigilance reported in the literature, we tested whether higher fNIRS global signals are associated with lower vigilance. The analysis has excluded the frequency band of greater than 0.05 Hz that contains the Mayer wave. Also, the analysis only considered the eyes-open condition to exclude the body position factor on EEG or fNIRS. Then, per each individual, the EEG vigilance at three body positions was sorted into highest, medium and lowest levels; and the fNIRS global signal associated with the highest vigilance levels were compared to fNIRS global signals at the medium or lowest level using a paired, two-sample and one-sided *t*-test. Furthermore, for the purpose of determining whether vigilance variations underlie the fluctuations in fNIRS global signal, the co-variation was assessed in one eye condition at one body position across all subjects, because practically resting state experiments are conducted in a single experiment condition rather than combined. Partial correlations between global signal amplitude and vigilance measures were calculated, by controlling age and gender as confounding factors.

While Experiment 1 focused on the stationary properties of fNIRS and EEG, we further evaluated whether the epoch-by-epoch fluctuations in fNIRS global signal and EEG vigilance are associated in Experiment 2. For each subject, we calculated the fNIRS global signal amplitudes and EEG vigilance measures in 30-s epochs. Then the temporal correlation between the fNIRS global signal and EEG vigilance was calculated using Pearson’s correlation coefficient across all epochs per each subject. To assess the temporal correlations at a group level, the correlation coefficients were converted to *z* scores using the Fisher’s transform. Afterward, a one-sample *t*-test against 0 was performed on all individuals’ z scores to evaluate the significance of temporal correlation at a group level.

## Results

The aim of the study was to investigate the neurological basis of the fNIRS resting-state global signal, if any, and the impact of body positions on the resting-state signals. The results are organized as such: In Experiment 1, the frequency-dependent impact of body positions on fNIRS and EEG signals was explored, then the factors of body positions and eye conditions were assessed in fNIRS global signal in two delineated frequency bands, and finally, the co-variation in the amplitude of fNIRS global signal and EEG vigilance was analyzed. As control results, the fNIRS and EEG task responses to auditory stimuli were included. In Experiment 2, the epoch-to-epoch fluctuations of fNIRS global signal amplitudes and EEG vigilance measures across 30-s epochs were examined and their temporal correlation was reported.

### Experiment 1: 10-Min Resting at Different Body Positions

**FIGURE 2:**
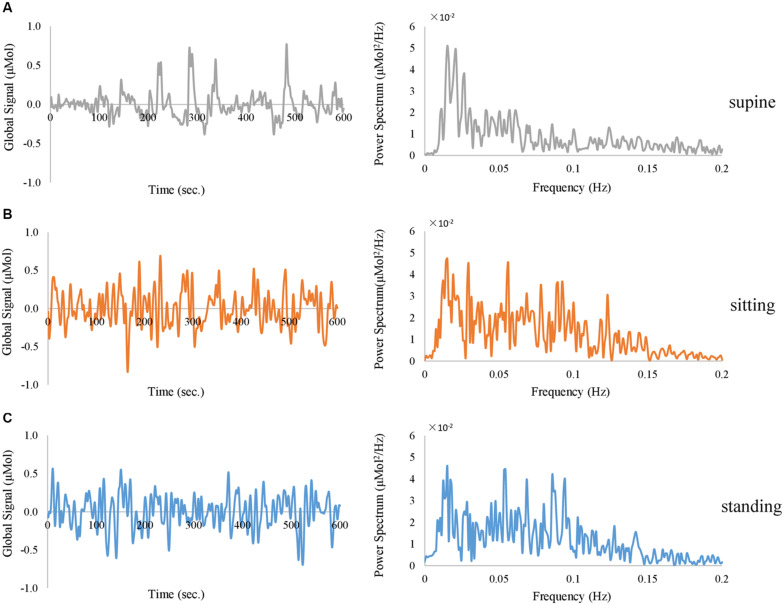
Representative traces of fNIRS global signal derived from HbO signal when the subject closed the eyes and rested in **(A)** supine position (in gray color), **(B)** sitting position (in orange color), and **(C)** standing position (in blue color), exhibited in the time domain (left panel) and frequency domain (right panel).

Firstly, spontaneous fluctuations were observed in the fNIRS global signal when subjects rested with their eyes open and closed, without any external stimuli. Representative single-session traces of fNIRS global signals are shown in Figure 2, at an EC resting condition. Notably, the global signal at all three positions exhibit fluctuations with a peak frequency of ∼0.02 Hz. Meanwhile, the data acquired from these body positions exhibited different patterns of fluctuations in the time domain, i.e., slower fluctuations are observed in the supine position and faster fluctuations in the sitting and standing positions. In terms of the amplitude, we noted that the power spectrum at the supine position showed a lowest amplitude in the frequency range of 0.05 – 0.1 Hz than those at sitting and standing positions, in the representative subject.

**FIGURE 3:**
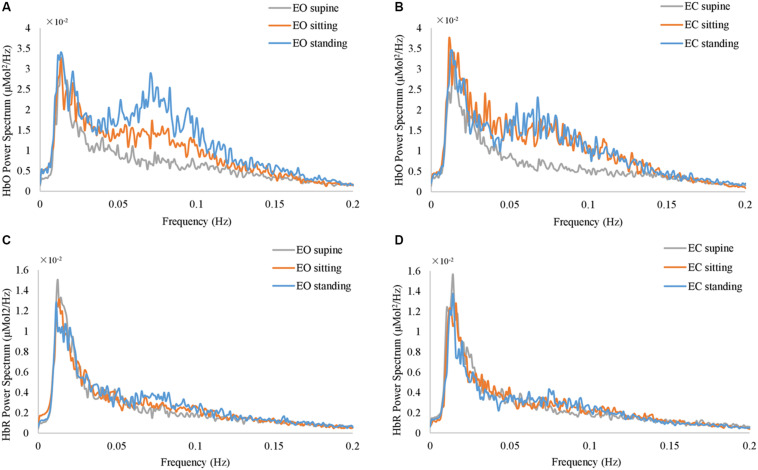
The grand average of the power spectrum of fNIRS global signals at resting state. **(A)** and **(B)** show HbO signal at EO and EC conditions. **(C)** and **(D)** show HbR signal at EO and EC conditions, respectively. The gray, orange, and blue curves represent supine, sitting, and standing position, respectively, in all panels.

**FIGURE 4:**
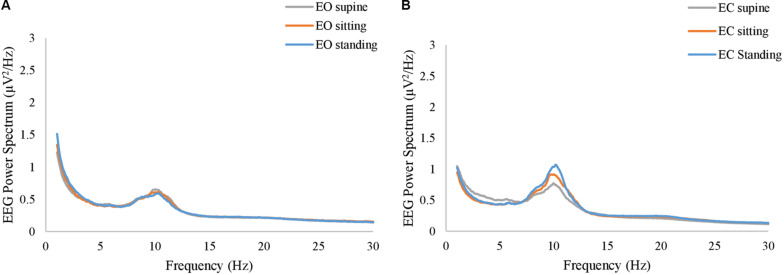
The grand average of the power spectrum of EEG resting-state signals at **(A)** EO condition and **(B)** EC condition. The gray, orange, and blue curves represent supine, sitting, and standing position, respectively, in both panels.

Furthermore, the position-dependent profile of the resting-state fNIRS global signal is also prominent at the group level. Figure 3 shows the grand average of the power spectrum of fNIRS global signal at various resting state conditions. Notably, in the frequency range from 0.05 Hz to 0.1 Hz, the spectrums at three different body positions show largely different amplitudes. The spectrum at the standing position appears to be of highest amplitudes in 0.05 – 0.1 Hz, at both EO and EC conditions (Figures 3A,B) in blue curves, whereas spectrum at supine are of lowest amplitudes (Figures 3A,B) in gray curves. In order to delineate the frequency-dependent effect of body position, we performed one-way ANOVA on the amplitude of fNIRS global signal separately in each frequency bin. At the EO condition, between 0.05 and 0.09 Hz, the effect of body position was significant on HbO (*p* < 0.05, uncorrected). Similarly, at the EC condition, the effect of body position was significant in the range from 0.07 to 0.09 Hz on HbO (*p* < 0.05, uncorrected). Since the fNIRS signal in the frequency range of 0.05 – 0.1 Hz has been related to a physiological noise known as the Mayer wave (Julien, 2006), our later analysis of the fNIRS global signal then focused on two distinct frequency bands, i.e., *f* < 0.05 and *f* > 0.05 Hz, in order to distinguish a position-dependent impact that may be attributed to physiological noises. In HbR (Figures 3C,D), we used the same frequency bands as with HbO. Noteworthy, none of HbO or HbR data showed any significant effect of body positions in a frequency bin less than 0.05 Hz (*p* > 0.05, uncorrected).

Likewise, in the resting-state EEG, our analysis explored whether a position-dependent profile exists on the spectrum. Figures 4A,B show the grand average of the power spectrum at EC and EO conditions, respectively. ANOVA revealed that the body position was *not* significant in any of the frequency bins at either EO or EC conditions (*p* > 0.05, uncorrected). Notably, although the grand average at the EC conditions appears with different amplitudes for three different conditions, it did not reach a significance level (*p* = 0.067 at *f* = 10.8 Hz, uncorrected).

Next, we aggregated the fNIRS and EEG quantities as the amplitude of global signal and vigilance scores, respectively. In particular, we averaged the amplitude of the fNIRS global signal (as root-mean-square) in the range of *f* < 0.05 Hz, which excludes the Mayer wave, and then separately in the range of *f* > 0.05 Hz. Meanwhile, EEG vigilance scores were calculated based on the power spectrum of resting state EEG as the ratio of alpha-band RMS divided by the sum of delta- and theta-band RMS. Two-way Repeated Measures ANOVA (body positions × eye conditions) revealed that the effect of body positions was *not* significant on fNIRS global signal amplitude in the very low frequency range of *f* < 0.05 Hz (*q* = 0.10). Meanwhile, the effect of body position was significant on the fNIRS global signal in the range of *f* > 0.05 Hz (*q* < 0.001). Noteworthy, the interaction of body positions and eye condition was *not* significant in fNIRS global signal amplitude in either frequency range.

**FIGURE 5:**
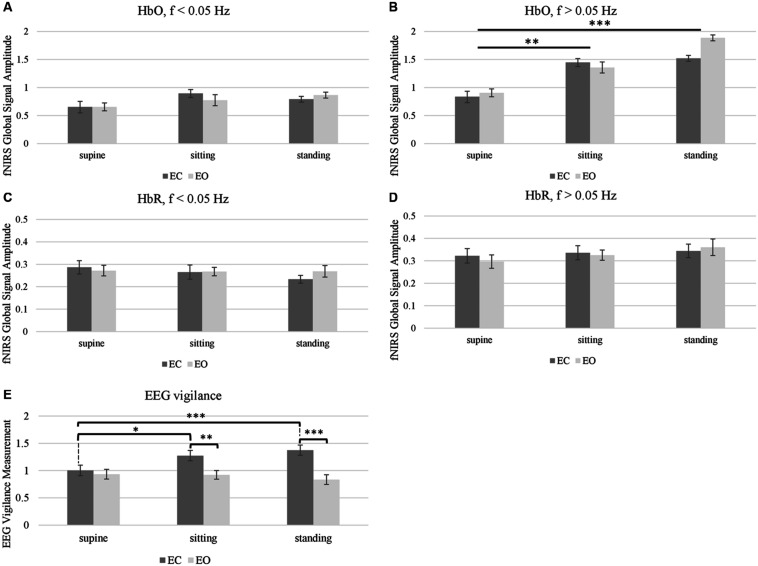
Effects of body positions (supine, sitting, and standing) and eye conditions (EC and EO) on **(A–D)** fNIRS global signal amplitude, and **(E)** EEG vigilance measurement. The effect of body position was significant in HbO global signal amplitude, in the range of *f* > 0.05 Hz that contains Mayer wave **(B)**. EEG vigilance measurement showed significance in the effect of body position, the effect of eye conditions and the position-eye interaction **(E)**. Stars indicate *post hoc* significance after multiple comparison correction (^∗^ indicates *q* < 0.05, ^∗∗^ indicates *q* < 0.01, ^∗∗∗^ indicates *q* < 0.001). Error bars indicate standard error.

*Post hoc* comparison on HbO in the range of *f* > 0.05 Hz was then conducted to assess the difference between pairs of body positions (i.e., standing vs. supine, sitting vs. supine, and standing vs. sitting) (Figure 5B). Analysis showed that the amplitude of fNIRS global signal for the supine position was significantly lower than the sitting position (*q* < 0.01) and standing position (*q* < 0.001), after multiple comparison correction. But amplitude of fNIRS global signal for sitting position did not differ from the standing position. Noteworthy, neither the eye factor or the eye-position interaction was significant in fNIRS HbO or HbR data.

In terms of EEG vigilance scores (Figure 5E), the two-way repeated measure ANOVA found that the effect of body position, the effect of eye condition and the eye-position interaction was all significant (*q* < 0.001). *Post hoc* comparisons on the EEG vigilance scores were then conducted to assess the differences. Informed by the significant interaction factor, we performed separate ANOVA analysis on the effect of body positions at separate eye condition and also performed separate *t*-test on pairs of body positions and eye conditions. Only under EC condition, the supine position had significant smaller EEG vigilance than sitting (*q* < 0.05) and standing position (*q* < 0.001). Furthermore, regarding the eye factor (EO vs. EC), the EEG vigilance showed significance at both sitting (*q* < 0.01) and standing positions (*q* < 0.001), but not in the supine position. However, under EO condition, there was no significant effect of body positions.

**FIGURE 6:**
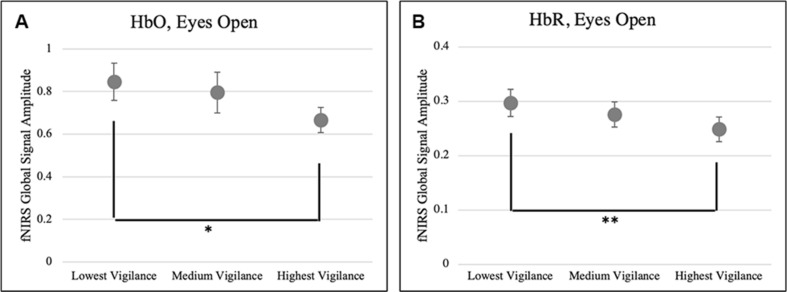
A reversed association between EEG vigilance and fNIRS global signals. Resting state sessions of lowest vigilance measures were associated with significantly higher amplitudes of fNIRS global signals in the **(A)** HbO (*p* < 0.05) and **(B)** HbR (*p* < 0.01). ^∗^*p* < 0.05, ^∗∗^*q* < 0.01, and error bars indicate standard error.

As a next step, we examined the relationship between the fNIRS global signal and the EEG at resting state. Firstly, we tested whether higher fNIRS global signals are associated with lower vigilance, which was motivated by a negative relationship between fMRI global signal and EEG vigilance reported in the literature. Particularly, we compared the amplitude of fNIRS global signal in the frequency range of *f* < 0.05 Hz against the EEG vigilance scores, only at EO state when either fNIRS or EEG quantities were *not* impacted by the factor of body positions. Results in Figure 6 shows a reversed association was identified between EEG vigilance and fNIRS global signals. After sorting the vigilance measures within each individual, the resting sessions of lowest vigilance were associated with significantly higher HbO global signals [*t*(18) = 2.02, *p* < 0.05] and also higher HbR global signals [*t*(18) = 2.98, *p* < 0.01] than those of highest vigilance. Importantly, note that neither the vigilance nor the global signal differed between body positions at the eyes-open condition; nonetheless, a reversed association was still found between EEG and fNIRS.

**FIGURE 7:**
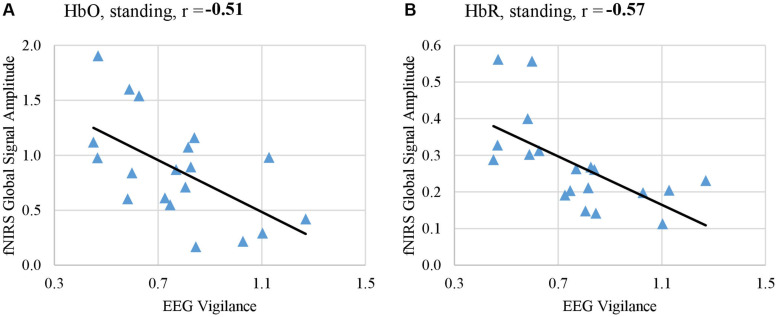
Amplitude of fNIRS global signal in the range of *f* < 0.05 Hz co-varied with EEG-based measure of vigilance, when subjects rested in the standing position with their eyes open. fNIRS global signal amplitudes derived from HbO signal **(A)** and from HbR signal **(B)** both significantly co-varied with EEG vigilance after controlling age and gender as confounding factors (HbO: *r* = –0.51, *p* < 0.05, HbR: *r* = –0.57, *p* < 0.05).

In addition, we examined the co-variation between fNIRS global signal and EEG per each body position across individuals. Results showed a consistent negative trend such that higher global signals are associated with lower vigilance states. In particular, both HbO and HbR at the standing position significantly co-varied with the EEG vigilance after controlling age and gender as confounding factor (HbO: *r* = −0.51, *p* < 0.05; HbR: *r* = −0.57, *p* < 0.05), as shown in Figure 7. However, at other positions, the covariation did not reach significance after multiple comparison correction, although a negative trend in the association was consistently noted. HbR at the supine position showed a significance-approaching covariation with EEG vigilance (*r* = −0.36, *p* = 0.1) and HbO at the sitting position also approached significance (*r* = –0.34, *p* = 0.1). HbO at supine position (*r* = −0.06) and HbR at sitting position (*r* = −0.20) did not reach a significant covariation with EEG vigilance.

**FIGURE 8:**
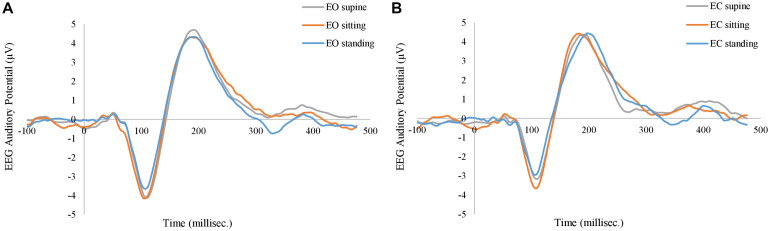
Grand average of EEG auditory evoked potentials at **(A)** eyes-open (EO) condition, and at **(B)** eye-closed (EC) condition. The gray, orange, and blue curves represent supine, sitting, and standing positions in both panels.

In order to control systematic and environmental nuisances, task-induced responses in EEG and fNIRS were investigated. Figure 8 shows the grand average of EEG AEP curves at three different body positions, from the FCz electrode. In either EO and EC conditions, the AEP curves at three body positions followed a very similar profile: negative activities at the ∼100 ms (N1) and positive activities at the ∼200 ms (P2). The factor of body positions has *no* significant effect on N1 or P2 (*q* > 0.1). Furthermore, the eye-position interaction is not significant, either (*q* > 0.1).

**FIGURE 9:**
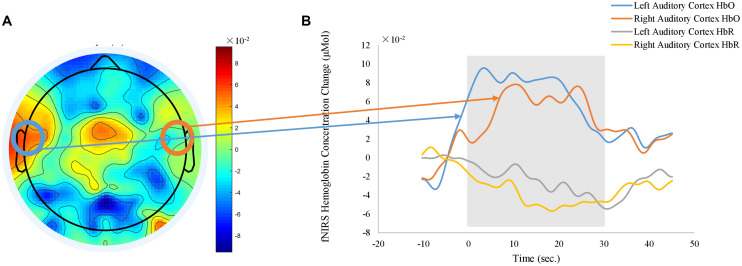
Grand average of fNIRS auditory response derived from HbO signals. **(A)** shows the topography of averaged HbO responses between 10 to 30 s after the stimulus onsite). **(B)** plots the time courses of fNIRS auditory response obtained from representative channels over left and right auditory cortex (orange: left auditory cortex HbO, blue: right auditory cortex HbO, gray: left auditory cortex HbR, yellow: right auditory cortex HbR). Shaded gray area indicates the time window of auditory stimulus. Time zero is the onsite of stimulus.

Meanwhile, task-related fNIRS responses were averaged across the blocks after subtracting the activities between −5 s and 0 s, with time 0-s as the beginning of the block. Figure 9 shows the grand average of fNIRS response to auditory stimuli. Representative time courses from the channels located over the left and right auditory cortex regions are shown in Figure 9B. When the auditory stimuli were on (shaded gray area in Figure 9B), relative changes of the HbO increased while the relative changes of the HbR decreased. In order to visualize the topography of auditory task responses, the block-average time series of fNIRS was selected from 10 to 30 s, averaged, and shown in Figure 9A. Positive activations of HbO are shown in left and right auditory cortex in the topography of HbO (Figure 9B). Results revealed *no* significant effect of body positions on the fNIRS auditory response.

### Experiment 2: 45-Min Resting at Supine Position

**FIGURE 10:**
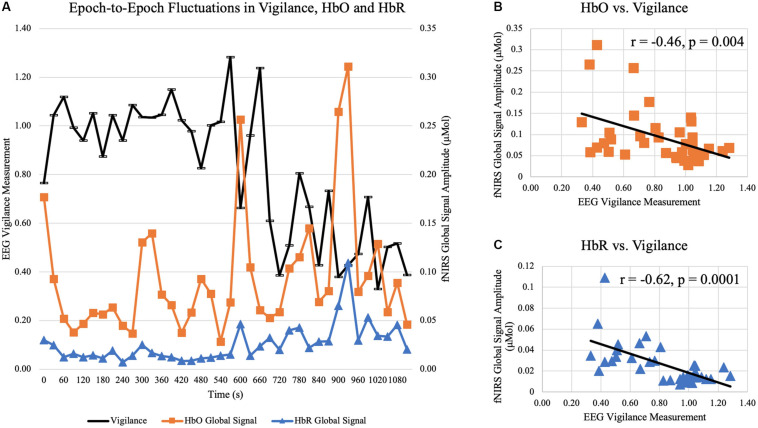
Temporal correlation between epoch-to-epoch fluctuations of vigilance and fNIRS global signal in a representative subject at wakeful rest. **(A)** plots the time courses of EEG vigilance measure (black curve) and global signal amplitude of HbO (orange curve) and HbR (blue curve) calculated in 30-s epochs over 18 min. **(B)** shows the negative correlation between vigilance and HbO global signal amplitude is significant (*r* = –0.46, *p* = 0.004). **(C)** shows the negative correlation between vigilance and HbR global signal amplitude is significant (*r* = –0.62, *p* < 0.001).

In Experiment 2, subjects were instructed to rest for a total duration of 45 min while allowed to fall asleep. Sleep scoring found that all 19 were able to fall asleep. Only data at wakeful resting before any sleep were used in the current analysis (Mean ± SD = 711 ± 602 s, ranging from 150 to 2430 s). After quantifying vigilance and fNIRS global signal amplitude in 30-s epochs, temporal fluctuations were observed. The time courses of HbO, HbR, and EEG vigilance measurement in a representative subject are displayed in Figure 10A. Every dot represents the global signal amplitude calculated from a windowed 30-s fNIRS signal and vigilance measurement calculated from 30-s EEG in the same aligned time window. As the subject gradually fell into the sleep, the vigilance exhibited a slowly decreasing trend, in reversed synchrony with surges of increases in fNIRS global signal and temporal. In the same time course, scattered moments of rebounds in vigilance are also aligned with drops of global signals, especially toward the later duration before the subject fell into sleep. In the same subject (shown in Figures 10B,C), a negative temporal correlation was observed between fNIRS global signal amplitude and EEG vigilance measurement (HbO vs. vigilance: *r* = −0.46, p = 0.004; HbR vs. vigilance: *r* = −0.61, *p* < 0.001).

At the group level, all subjects exhibited temporal variations in EEG vigilance. Specifically, the standard derivation of EEG vigilance over the period of wakeful rest ranged from 0.07 to 0.46 across all subjects (Mean ± SD = 0.25 ± 0.13). In terms of association, analysis showed that there existed significant correlation between HbO global signal amplitude and vigilance [*t*(19) = −2.57, *p* = 0.02), as well as significant for HbR and vigilance [*t*(19) = −2.80, *p* = 0.01]. Despite that subjects had different durations of wakefulness, the window length of wakefulness was not associated with the temporal correlation between fNIRS global and vigilance (*p* > 0.1 for HbO and HbR). When restricting the wakeful epochs to be within the 10 min before falling into sleep, the temporal correlation was still significant between HbO and vigilance [*t*(19) = −2.32, *p* = 0.03], and also significant between HbR and vigilance [*t*(19) = −2.83, *p* = 0.01]. Furthermore, considering that individuals exhibited different levels of vigilance fluctuations (i.e., standard derivation of EEG vigilance ranged from 0.07 to 0.46), we examined whether the standard derivation of EEG vigilance modulated the association between EEG vigilance measures and fNIRS global signal; yet the analysis showed that the scale of vigilance fluctuation levels was not relevant (*p* > 0.1).

## Discussion

Our study has investigated the neurophysiological nature of the global signal of fNIRS measured at resting state. The results for the first time have demonstrated that the amplitude of the fNIRS global signal, particularly in the frequency range of 0.01 < *f* < 0.05 Hz, is reversely associated with EEG vigilance measures. The discovery of a neurological origin for fNIRS global signal has important implications for the processing of fNIRS signal acquired at resting state.

One of the most fundamental and critical issues in analyzing neuroimaging data is how to handle the global signal, which is defined as the time series of intensity averaged across imaging units in PET (Fox et al., 1988; Friston et al., 1990), fMRI (Desjardins et al., 2001; Macey et al., 2004), and more recently in fNIRS (Franceschini et al., 2006; Zeff et al., 2007; White et al., 2009; Mesquita et al., 2010). A strong presence of global signal in fMRI may lead to a massive and diffused activation pattern in task-based studies if the time series of the global signal is of similar profile with the task modulation (Kay et al., 2013; Power et al., 2015). Likewise, fNIRS studies of various tasks commonly removed a global component derived from the measurements to reveal focal activations, via linear regression or spatial filtering based on PCA/ICA decomposition (Zhang et al., 2005, 2007, 2016; Franceschini et al., 2006; Kohno et al., 2007; Zeff et al., 2007; Eggebrecht et al., 2012, 2014; Sato et al., 2016). Nonetheless, the impact of global signal is more problematic in task-free, resting state studies, as the global signal may lead to a perfusive connectivity pattern that is attributed to the global signal, no matter whichever seed region of interest is selected. Because region-specific connectivity is more desirable and because non-neuronal sources can dominantly contribute to the global signal (Glover et al., 2000; Wise et al., 2004; Birn et al., 2006; Yuan et al., 2013), the analysis of resting state fMRI data has commonly included steps to attenuate the impact of a global signal. For example, GSR removes an averaged signal of all recording units from the time series through linear regression. This procedure was originally developed for and applied to task-based fMRI data (Zarahn et al., 1997; Aguirre et al., 1998; Macey et al., 2004). Later, most resting-state fMRI studies have adopted GSR as a pre-processing approach: the global signal component is regressed out of preprocessed BOLD signals prior to computation of connectivity measures and therefore regionally focused connectivity patterns are reported (Fox et al., 2009). Similarly, in recent fNIRS studies of resting state brain, a global component has been recognized in the measurements from regularly distanced optodes (White et al., 2009; Mesquita et al., 2010; Tong and Frederick, 2010; Eggebrecht et al., 2014; Tachtsidis and Scholkmann, 2016; Duan et al., 2018; Wyser et al., 2020) and from short-distanced optodes (White et al., 2009; Gregg et al., 2010; Mesquita et al., 2010; Eggebrecht et al., 2014; Tachtsidis and Scholkmann, 2016; Duan et al., 2018; Sherafati et al., 2020; Wyser et al., 2020). To date, there is no well-established pre-processing routine in resting state fNIRS studies although multiple efforts are being made (Huppert et al., 2009; Ye et al., 2009; Xu et al., 2014; Santosa et al., 2018). Approaches such as GSR and spatial filtering via PCA and ICA decomposition that were used in task-based fNIRS studies are also commonly adapted in resting state fNIRS studies to remove the global component, yielding regionally focused connectivity pattern (Mesquita et al., 2010; Zhang et al., 2010, 2011; Sakakibara et al., 2016).

However, the removal of global signal in neuroimaging data has encountered controversial critiques, particularly in the studies of resting state functional connectivity. Because a global neurophysiological component may be present in direct neural recordings (Scholvinck et al., 2010; Wong et al., 2013, 2016), removing the global signal is shown to cause loss of such neural components, thereby confounding the resulted pattern of resting state functional connectivity. For example, Chen et al. (2012) found that the global signal is highly correlated with DMN component. Further evidences indicated that the global signal resembles the resting-state fMRI time courses of the largest cluster when the level of global noise is low (Chen et al., 2012). Under such circumstances, GSR could mathematically mandate the presence of anti-correlation network in fMRI studies (Murphy et al., 2009). Other studies have further linked the fluctuations of global signals to the varying levels of vigilance or arousal (Chang et al., 2016; Falahpour et al., 2018), which suggests that removing the global signal in those situations could remove an underlying behavioral factor. Therefore, the GSR should be very carefully applied when studying resting-state MRI (Murphy et al., 2009; Saad et al., 2012; Murphy and Fox, 2017). Until now, the nature of the fNIRS global signal has not been fully established since the neurophysiological components in the resting-state global fNIRS signal have not been systematically investigated. Our current study is the first of its kind to investigate the neuronal and non-neuronal sources in the fNIRS global signal by using concurrent fNIRS and EEG in whole-brain and high-density setup. Because both fNIRS and BOLD fMRI measure the cerebral hemodynamics, they carry similar substrates for neuronal activities while they also share common caveats due to non-neuronal sources, including respiration, cardiac pulsations, motion, etc. Like in the case of fMRI, removal of fNIRS global signal may lead to spurious results in the functional connectivity pattern, depending on whether or not there exists any neural component in the global signal of fNIRS and the amplitude level of global signal.

In this study, we have shown that fNIRS global signals acquired from the resting human brain are periodical oscillations. As shown in Figures 2, 3 at respective individual and group level, the resting-state fNIRS global signal resides in three ranges: dominantly less than 0.05 Hz with a peak component at ∼0.02 Hz, a second peak between 0.05 and 0.1 Hz (also known as the Mayer wave) and greater than 0.1 Hz. Furthermore, our study extended investigations of the fNIRS global signal at standing, sitting and supine positions. Indeed, periodic fluctuations were observed in the global signal at all body positions. The presence of a fluctuating fNIRS global signal with dominate activities of <0.1 Hz suggests that the RSFC pattern may be affected by the global signal. Comparing with intracranial neural recordings (Leopold et al., 2003; He et al., 2008; Shmuel and Leopold, 2008), fNIRS global signal and spontaneous neural activities overlap their peaks in the range of <0.1 Hz. Meanwhile, in comparison with fMRI, the fNIRS global signal shows a very similar spectral profile with those from BOLD fMRI. Especially, the spectrum of fNIRS at the supine position (Figures 3A,B) for both EO and EC conditions are almost identical to those reported in fMRI (e.g., Figure 1 in Biswal et al., 1995). Since in our study the whole-head fNIRS montage were sampled at 1.95 Hz, which is a higher frequency than BOLD fMRI (usually 0.5 Hz), the spectrum of fNIRS global signal revealed a more accurate spectrum.

Importantly, for the first time our study reported a negative association between the amplitude of fNIRS global signal in the range of <0.05 Hz and the EEG vigilance based on the simultaneous recording in the Experiment 1 (Figures 6,7). Within individuals, the resting state sessions with lowest EEG vigilance measures were associated with significantly higher fNIRS global (HbO and HbR in Figure 6), which was observed at eyes open condition and neither EEG nor fNIRS was affected by body positions. Furthermore, in a single body position at eyes-open condition, a negative covariation between fNIRS global signal amplitude and EEG vigilance was also confirmed across individuals (HbO and HbR in Figure 7). The selection of frequencies *f* < 0.05 Hz for fNIRS is critical: it is within the range of resting state fMRI data but distinctly narrower to exclude the Mayer wave. Previous fMRI study has demonstrated that the functional connectivity in auditory, visual and sensorimotor cortices is characterized 90% by the low-frequency band from 0 to 0.1 Hz (Cordes et al., 2001). Meanwhile, the fractional amplitude of low-frequency fluctuation (fALFF) is defined as the ratio of power spectrum of 0.01 – 0.08 Hz to that of the whole frequency band (Zou et al., 2008). Noteworthy, one of the most studied networks – DMN – has significantly higher fALFF than other brain regions, which indicates DMN has higher intensity of regional spontaneous brain activity in the range of 0.01 – 0.08 Hz (Zou et al., 2008). More importantly, our fNIRS signal was further narrowed to the range of <0.05 Hz, in order to avoid the Mayor wave which is shown to depend on body positions. Because of a high sampling frequency, fNIRS was effective in preventing aliasing of high-frequencies related to pulse and respiration into the range of <0.05 Hz.

In addition, our results revealed that the power spectrum of HbO global signal depends on body positions in the range between 0.05 – 0.1 Hz, regardless eyes were opened and closed (shown in Figures 2, 3 at respective individual and group level). Data at the standing position show the largest amplitude than the others, while the supine position is associated with lowest amplitude. These findings are consistent with previous reports by Tachtsidis et al. (2004), who compared three different positions’ effect on cerebral blood pressure with fNIRS. Their results showed that standing position has the highest mean blood pressure (MBP) and supine has the lowest MBP. They followed the Task Force of the European Society of Cardiology and the North American Society of Pacing and Electrophysiology (1994) to separate the frequency spectrum into three standard frequency bands: very low frequency (VLF: 0.02–0.04 Hz), low frequency (LF: 0.04–0.15 Hz) and high frequency (HF: 0.15–0.4 Hz). Although VLF did not reveal any significant impact of body position, their results reported that the magnitude of low frequency oscillation in HbO in the resting brain shows a significant difference between different postures in LF. Coincidentally, Mayer wave, i.e., the cyclic changes in arterial blood pressure, fall into this LF range (Muller et al., 2003; Julien, 2006). Mayer wave appears to have a close relationship with fNIRS global signal. It is observed as oscillations of arterial pressure at ∼0.1 Hz in conscious humans (Julien, 2006). Besides, it is positively related with the strength of the corresponding sympathetic nervous activity and the mean level of sympathetic nerve activity (Furlan et al., 2000). More importantly, prone, supine, and sitting have significantly different effect on autonomic regulation of cardiovascular function (Watanabe et al., 2007). One rational speculation is that different body positions, especially the up-tilt positions, significantly affect autonomic regulation includes SNA which set the level of sympathetic vasoconstrictor tone, hence contributing to sustain arterial pressure (Julien, 2006; Scholkmann et al., 2014; Mohammadi-Nejad et al., 2018). Therefore, we regarded position-dependent effect in the Mayer wave range to be of physiological origin and discarded them for comparison against EEG. Aside from the Mayer wave range, our analysis further eliminated the factor of body positions and revealed a negative association between the EEG vigilance and fNIRS global signal in the frequency range of <0.05 Hz (HbO and HbR in Figures 6, 7). Such EEG-fNIRS association for the first time revealed a neurophysiological contribution to the fluctuations of fNIRS global signal (due to EEG vigilance), rather than a physiological factor (due to body positions). As control data in the Experiment 1, we conducted qualitative analysis and statistical analysis on auditory EEG and fNIRS responses. Our results did not observe the different body positions’ effect on AEP of EEG data or task-related average of fNIRS data, at both EO and EC conditions. This excludes the concerns of environmental and systematic biases, such as the quality of data recording when subjects were positioned differently.

Our findings of a negative association between fNIRS global signal and EEG vigilance measures have important implications for the analysis and interpretation of fNIRS-based resting state functional connectivity. The reversed association between EEG vigilance and fNIRS global signal observed within individuals (Figure 6) indicates that removal of fNIRS global signal will also remove the neurological effect of vigilance in the signals. Therefore, in resting state functional connectivity studies using repeated fNIRS measures within an individual, especially if the subjects’ conditions are related to the vigilance levels, global signal removal should not be performed. In addition, a negative covariation across individuals at the standing position (Figure 7) indicates that removal of global signal will also remove the neurological effect of vigilance in the signals. Therefore, in resting state studies using fNIRS (such as biomarkers of disease across individuals), especially when vigilance is an individual-level trait relevant to disease symptoms (Yang et al., 2017) or behaviors (Li et al., 2019), global signal removal could become problematic.

Furthermore, our study in the Experiment 2 for the first time reported a negative temporal correlation between the epoch-by-epoch fluctuations of fNIRS global signal and EEG vigilance, which further confirmed the negative association observed across subjects in Experiment 1. In wakeful rest periods that were verified by sleep scoring criteria, subjects exhibited momentary upsurges and drops of vigilance, as they stayed awake but were falling into sleep. Such vigilance fluctuations were then shown to be in reversed synchrony with the HbO and HbR global signal: epochs of decreased vigilance were associated with surges in fNIRS global signal, and vice versa. Interestingly, such findings of negative association are consistent with other studies that have examined the BOLD fMRI signals and EEG or behavior measures of vigilance. For example, Olbrich et al. (2009) of simultaneous EEG and fMRI study have reported that decreased of EEG vigilance measures are associated with a perfusive increase of BOLD signals in widespread cortices (except the thalamus). Similarly, Liu and colleges have observed a negative temporal correlation between EEG vigilance and fMRI global signal calculated from whole-brain average, which was reported in 23 sessions out of the 25 sessions in total and ranged between 0 and −0.5 (Falahpour et al., 2018, Figure 4), which is similar with our observation. In another study performed in unanesthetized monkeys, Chang et al. (2016) investigated the behavior measure of vigilance, indicated as opening and closure of eyes, and reported again that the fluctuations of vigilance level have negative temporal correlation with BOLD signals in widespread cortices, in a similar spatial extent and consistent temporal manner with those observed in human studies (Olbrich et al., 2009; Falahpour et al., 2018). Our study, however, reported for the first time the negative temporal correlation exists in concurrent and whole-head fNIRS and EEG recordings in human. Our findings of the epoch-by-epoch association are important for the interpretation of dynamic resting state functional connectivity, which commonly used a windowed approach of 30-s to 120-s duration. While the resting state brain connectivity is increasingly recognized to possess rich information of dynamics (Hutchison et al., 2013), some studies removed the global signal (Allen et al., 2014) whereas other studies did not (Chang et al., 2013). By showing that the fNIRS global signal amplitude is negatively correlated with EEG vigilance, our findings suggest that the removal the global signal should not be performed in the investigation of dynamic connectivity using fNIRS, especially in conditions affected by vigilance fluctuations. Beyond that, DMN has been reported to be correlated with EEG vigilance scores (Olbrich et al., 2009). Removing fNIRS global signal therefore may attenuate activities of DMN that are related with vigilance fluctuations. Evidence has shown that the working memory plays a critical role in both visual rehearsal and vigilance performance (Baddeley et al., 1999). And age-related alterations and disease-related decrements (such as Alzheimer’s disease) in DMN have significantly impacted working memory performance (Baddeley et al., 1999; Sambataro et al., 2010). Therefore, the fNIRS global signal should not be treated as non-neural confound, and its removal should be carefully considered via a frequency delineation.

Noteworthy, the calculation of the fNIRS global signal amplitude in our study is a reasonable adaption from the definition of global signal amplitude in previous fMRI study (Wong et al., 2013). Considering that the fNIRS optical density is converted to relative changes of HbO/HbR concentration in the stage of hemodynamic computation, the normalization in fNIRS equates the normalization in fMRI analysis (i.e., divided by the mean of fMRI time course), the calculation of fNIRS global signal in our study followed exactly the same definition in Wong et al. (2013). Our findings are consistent with previous findings on the relationship between fMRI global signal and EEG vigilance (Wong et al., 2013, 2016). Such discovery of a neurological component in fNIRS global in our study is novel. Importantly, our investigation adds findings from a unique perspective by showing a covariation relationship in a carefully constrained frequency range that has excluded the possible physiological noise of blood pressure regulation. Our studies of two experiment datasets have demonstrated the reversed association exiting in both static and dynamic manner.

Additionally, it is worthy to note that the quantification of EEG vigilance has limitations, due to interindividual variance in EEG activity that is commonly observed in many EEG studies (Jobert et al., 1994; de Munck et al., 2007; Olbrich et al., 2009). For example, certain subjects may exhibit almost no EEG alpha peak in the power spectrum of resting EEG at eyes open and sometimes, even at eyes closed states. In the meanwhile, there are subjects that show strong alpha peak power at both eyes open and eyes closed states. Here in our analysis we have taken multiple steps to mitigate the factor of interindividual variance in EEG activity. Firstly, we have calculated a normalized spectrum of EEG accounting all frequency bins; and the vigilance measures was calculated as the ration between the alpha-band amplitude divided by the sum of amplitudes in the delta and theta bands based on the normalized spectrum. Then, in Experiment 1, we accounted the interindividual variance by contrasting between the lowest vigilance condition and higher vigilance conditions within individuals, at which subjects’ eyes were all open. Moreover, in Experiment 2, we examined the temporal fluctuations to determine the association between EEG vigilance and fNIRS, while we concluded that the levels of vigilance fluctuations did not affect the temporal association. Nonetheless, because we also examined the across-individual covariation (Figure 7), the observation of the association between EEG vigilance and fNIRS global signal could be attributed to interindividual variance in EEG activities rather than the neurological factor of vigilance, although removal the global signal under such situation could still introduce confounds to the fNIRS resting state functional connectivity analysis.

## Conclusion

With the advantage of economic efficiency and portability, fNIRS has been proposed as a complementary option to fMRI, especially to be used in populations with contraindications to MRI scanner and in challenged environment (such as brain monitoring at bed-side or during surgery). The current study for the first time revealed a negative relationship between fNIRS global signal amplitudes and EEG vigilance in human participants, based on concurrent EEG and fNIRS recordings at high-density and whole-head montage. Our results stressed the significant effect of body positions on the fNIRS resting-state global signal, primarily in the frequency range of greater than 0.05 Hz yet not in the range of less than 0.05 Hz. However, EEG vigilance plays a modulatory role in the fNIRS signals in the frequency range of less than 0.05 Hz: resting-state sessions of low EEG vigilance measures are associated with high amplitudes of fNIRS global signals. Moreover, the epoch-by-epoch fluctuations of EEG vigilance and fNIRS global signals are significantly correlated in a negative manner at a wakeful resting period. The findings of a neural component, i.e., EEG vigilance, in fNIRS global signal suggests that such global signal should not be removed as non-neural physiological signal, especially in studies and conditions where vigilance and related brain networks are of interest.

## Data Availability Statement

The raw data supporting the conclusions of this article will be made available by the authors, without undue reservation.

## Ethics Statement

The studies involving human participants were reviewed and approved by the Institutional Review Board at the University of Oklahoma Health Sciences Center. The patients/participants provided their written informed consent to participate in this study.

## Author Contributions

YC contributed to data collection, the data analysis, and manuscript writing. JT, YC, and MC contributed to data collection. JF and BC contributed to data analysis. HY contributed to study design, the data analysis, and the manuscript writing. All authors contributed to the article and approved the submitted version.

## Conflict of Interest

The authors declare that the research was conducted in the absence of any commercial or financial relationships that could be construed as a potential conflict of interest.
